# A high-density and high-confinement tokamak plasma regime for fusion energy

**DOI:** 10.1038/s41586-024-07313-3

**Published:** 2024-04-24

**Authors:** S. Ding, A. M. Garofalo, H. Q. Wang, D. B. Weisberg, Z. Y. Li, X. Jian, D. Eldon, B. S. Victor, A. Marinoni, Q. M. Hu, I. S. Carvalho, T. Odstrčil, L. Wang, A. W. Hyatt, T. H. Osborne, X. Z. Gong, J. P. Qian, J. Huang, J. McClenaghan, C. T. Holcomb, J. M. Hanson

**Affiliations:** 1 General Atomics, San Diego, CA USA; 2 Lawrence Livermore National Laboratory, Livermore, CA USA; 3 University of California San Diego, La Jolla, CA USA; 4 Plasma Science and Fusion Center, Massachusetts Institute of Technology, Cambridge, MA USA; 5 Princeton Plasma Physics Laboratory, Princeton University, Princeton, NJ USA; 6 Institute of Plasma Physics, Chinese Academy of Sciences, Hefei, China; 7 Department of Applied Mathematics and Applied Physics, Columbia University, New York, NY USA

**Keywords:** Magnetically confined plasmas, Nuclear fusion and fission

## Abstract

The tokamak approach, utilizing a toroidal magnetic field configuration to confine a hot plasma, is one of the most promising designs for developing reactors that can exploit nuclear fusion to generate electrical energy^1,2^. To reach the goal of an economical reactor, most tokamak reactor designs^3–10^ simultaneously require reaching a plasma line-averaged density above an empirical limit—the so-called Greenwald density^11^—and attaining an energy confinement quality better than the standard high-confinement mode^12,13^. However, such an operating regime has never been verified in experiments. In addition, a long-standing challenge in the high-confinement mode has been the compatibility between a high-performance core and avoiding large, transient edge perturbations that can cause very high heat loads on the plasma-facing-components in tokamaks. Here we report the demonstration of stable tokamak plasmas with a line-averaged density approximately 20% above the Greenwald density and an energy confinement quality of approximately 50% better than the standard high-confinement mode, which was realized by taking advantage of the enhanced suppression of turbulent transport granted by high density-gradients in the high-poloidal-beta scenario^14,15^. Furthermore, our experimental results show an integration of very low edge transient perturbations with the high normalized density and confinement core. The operating regime we report supports some critical requirements in many fusion reactor designs all over the world and opens a potential avenue to an operating point for producing economically attractive fusion energy.

## Abstract (web summary)

A stable tokamak plasma has been demonstrated with a high plasma density and a high energy confinement quality, both of which are simultaneously important for fusion reactors.

## Main

Fusion energy is the ultimate energy source for humanity^16^. A promising approach is a steady-state fusion reactor using magnetic confinement in the tokamak configuration^17,18^. With a deeper understanding of tokamak plasma physics and the development of reactor-relevant technologies, many fusion reactor designs have been proposed^3–10^. When the ion temperature is above 13 keV (1.5 × 10^8^ K) in D–T fusion reactions, the thermonuclear power density^19^ *P*_fus_ = *n*_fuel_^2^⟨*σv*⟩*E*/4 is proportional to the fuel density (*n*_fuel_) squared, as the change of normalized reaction rate ⟨*σv*⟩ with temperature is relatively small. Here, *E* is the fusion energy released per reaction. Detailed definitions of all variables mentioned in this paper can be found in Extended Data Table 1. Therefore, to achieve attractive fusion goals, most of the recent fusion pilot plant (FPP) designs require very high plasma densities, higher than the empirical edge density limit known as the Greenwald density^11^ (*n*_Gr_), in tokamak high-confinement mode (H-mode) plasmas^13^. The energy confinement quality, represented by the H-factor^20^ (for example, *H*_98y2_), is believed to be the highest leverage parameter for fusion capital cost^8^. *H*_98y2_ is usually required to exceed the standard H-mode level (*H*_98y2_ = 1.0) for good fusion economy. FPP designs^3–10^ simultaneously require 1 ≤ Greenwald fraction (*f*_Gr_) ≤ 1.3 and 1 ≤ *H*_98y2_ ≤ 1.65. However, such a tokamak operating regime is an uncharted area that has never been verified in experiments.

The empirical *n*_Gr_ is a density limit for the pedestal density in an H-mode plasma^21,22^. The pedestal is a narrow region of plasma at the edge with suppressed turbulent transport and a steep pressure gradient. When approaching *n*_Gr_ at the pedestal, various unfavourable phenomena can be observed in experiments. These cause a strong decrease of the confinement quality or even a sudden, complete loss of plasma energy (disruption)^22^. A peaked core density profile is, therefore, required to achieve a line-averaged density above the pedestal density limit. Possible approaches include relying on the natural peaking at low collisionality^23^ and the potential inward particle pinch^24^. The previous DIII-D experiment^24^ can achieve a transient *f*_Gr_ of about 1.4 with D_2_ gas puffing. A large pinch velocity has been measured. *H*_98y2_ in this case is around 1. ASDEX Upgrade experiments took a different approach by using pellet injection to improve the core fuelling. The experimental results show a transient *f*_Gr_ ≈ 1.5 with pellet injection^25,26^. However, the *H*_98y2_ values in those discharges were less than 1. More examples with *H*_98y2_ < 1 at high density are well documented^22^. As no tokamak experiment has yet attained a sustained *f*_Gr_ above 1 and *H*_98y2_ well above 1 (for example, 1.5) at the same time, experimentally verifying the desired operating regime in FPP designs is a great challenge for the magnetic confinement fusion community.

Another challenge with H-mode reactor plasmas is the very high transient heat load produced by quasi-periodic edge magnetohydrodynamic (MHD) instabilities known as type-I edge-localized-modes (ELMs). Without control, ELMs in a reactor can severely damage plasma-facing-components, for example, the first wall^27,28^. ELM control is an active research area and various approaches have been proposed^29–33^. However, compatibility among small/no ELM solutions, high density (above *n*_Gr_) and high confinement quality (*H*_98y2_ well above 1, for example, 1.5) has not been demonstrated in experiments.

We report a new experimental approach for achieving a line-averaged density above *n*_Gr_. It exploits an operating regime recently established in the DIII-D tokamak that allows simultaneous *f*_Gr_ > 1.0, *H*_98y2_ ≈ 1.5 and small ELMs and could support many existing designs for future reactors^3–10^. The approach elevates the plasma density in the core while keeping the pedestal fraction of the Greenwald density at moderate levels (for example, *f*_Gr,ped_ ≈ 0.7), thus not violating the empirical density limit. It does so by exploiting self-organized internal transport barriers (ITBs) at large minor radius in the high poloidal-beta (*β*_P_) scenario^15,34–36^. More information about the high-*β*_P_ research can be found in Methods. In experiments, the on-axis fraction of the Greenwald density (*f*_Gr,0_) can reach up to 1.7, resulting in a line-averaged *f*_Gr_ of 1.3. ITBs in the density and temperature profiles also greatly improve the energy confinement quality (*H*_98y2_ up to 1.8), compared to the standard H mode (*H*_98y2_ = 1) at the same engineering and operating parameters.

**Fig. 1:**
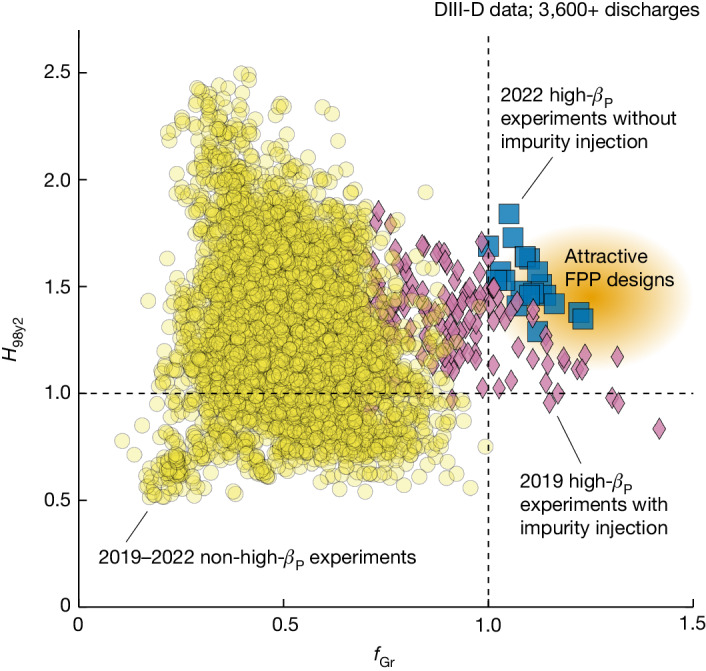
Database of *H*_98y2_ and *f*_Gr_ for DIII-D discharges. More than 3,600 discharges are included. Violet diamonds show high-*β*_P_ experiments performed in 2019 with impurity injection. Blue squares are the new high-*β*_P_ experiments performed in 2022 without impurity injection. Yellow circles represent all other experiments performed in 2019–2022. The area shaded in orange indicates the parameter space for attractive FPP designs. Vertical and horizontal dashed lines show *f*_Gr_ = 1.0 and *H*_98y2_ = 1.0, respectively.

**Fig. 2:**
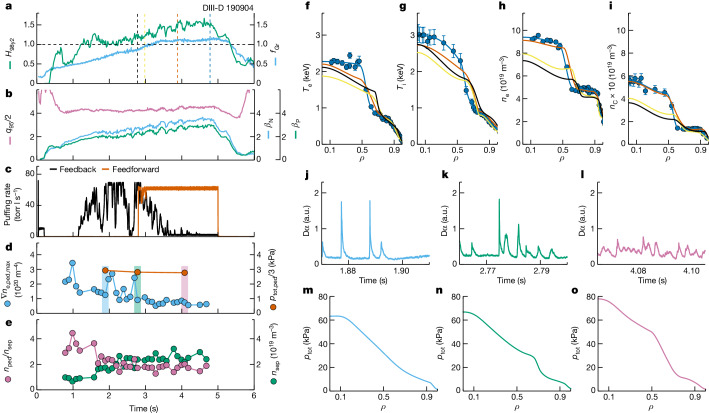
Time history of experimental parameters and plasma profiles of DIII-D 190904. **a**, *f*_Gr_ in blue and *H*_98y2_ in green. **b**, *β*_N_ in blue, *β*_P_ in green and *q*_95_ in violet. **c**, D_2_ gas puffing in feedback control in black and dedicated feedforward D_2_ gas puffing in vermillion. **d**, Peak pedestal electron density gradient in blue and pedestal total pressure in vermillion. **e**, Separatrix electron density in green and ratio between pedestal electron density and separatrix electron density in violet. **f**–**i**, Profiles of electron temperature (**f**), ion temperature (**g**), electron density (**h**) and carbon density (**i**) at the time slices shown in the vertical dashed lines in **a**. Dots with error bars are measurements. **j**–**l**, D*α* data for the three periods shown in the shaded area in **d**. a.u., arbitrary units. **m**–**o**, Total pressure profiles at the time slices of the vermillion dots in **d**.

Figure 1 shows a plot of the DIII-D database and illustrates the progress made in extending the plasma operating space towards high *f*_Gr_ and high *H*_98y2_. The 2019 high-*β*_P_ experiments with impurity injection^15^ have simultaneously achieved *f*_Gr_ > 1.0 and *H*_98y2_ > 1.0. However, in these experiments, too much impurity injection also increases the radiative energy loss in the plasma core, limiting *H*_98y2_ at high density. Of the violet diamonds in Fig. 1, some have *H*_98y2_ ≤ 1.2 when *f*_Gr_ ≥ 1.15. However, these results are not good enough for FPP designs. A major improvement in the 2022 DIII-D high-*β*_P_ experiment used additional D_2_ gas puffing (Fig. 2) instead of impurity injection. This approach effectively reduces the core radiation and improves *H*_98y2_, as shown in Fig. 1 (blue squares). Thus, this paper reports a clear experimental demonstration of an accessible operating point in an existing tokamak that can meet a few of the FPP requirements, including simultaneous *f*_Gr_ > 1 and *H*_98y2_ ≈ 1.5. For comparison, other scenarios presented run on DIII-D have not achieved such simultaneous normalized performance (yellow circles).

**Extended Data Fig. 1:**
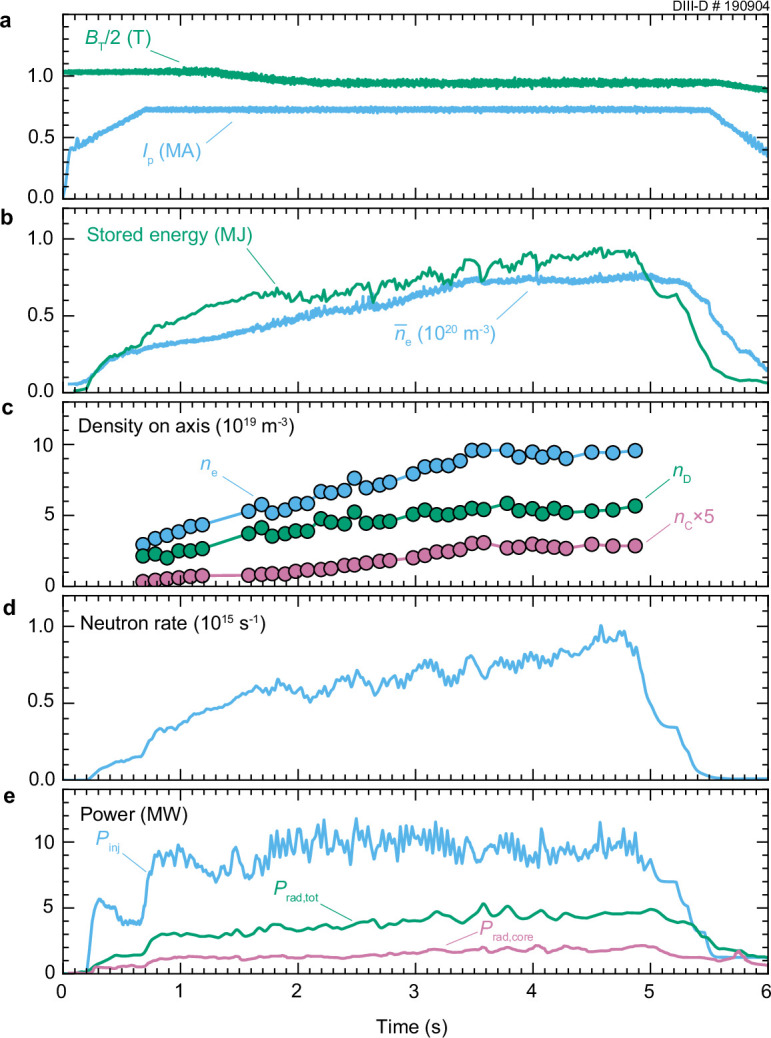
Additional time histories for DIII-D # 190904. (a) Plasma current in blue and toroidal field in green; (b) Line-averaged density in blue and stored energy in green; (c) On-axis electron density in blue, on-axis deuterium density in green and on-axis carbon density in violet; (d) Measured neutron rate; (e) Injected NBI power in blue, measured total radiated power in green and core radiated power in violet.

Figure 2 shows detailed data from an example discharge (190904) in 2022. The striking feature in this discharge is the dynamic synergy between energy confinement quality and plasma density. That is, *H*_98y2_ increased along with *f*_Gr_ (Fig. 2a) until the ramping down of the heating power (Extended Data Fig. 1e). This is opposite to the common observation of reduced energy confinement quality in higher density H modes^22^, especially for experiments close to the Greenwald density. The plasma was maintained at *f*_Gr_ > 1.0 and *H*_98y2_ > 1.0 for about 2.2 s, which was 2.2 times the current diffusion time (*τ*_R_) or 24 times the energy confinement time (*τ*_E_). Thus, the high normalized density and confinement phase was not transient, which is imperative for application in future long-pulse FPPs. A normalized plasma pressure *β*_N_ ≈ 3.5 and *β*_P_ ≈ 2.9 was achieved at safety factor *q*_95_ ≈ 8.5 (Fig. 2b) with plasma current *I*_p_ = 0.73 MA and toroidal magnetic field *B*_T_ = 1.89 T, and with mixed co- and counter-*I*_p_ neutral beam injection (NBI). Note that *n*_Gr_ = 6.7 × 10^19^ m^−3^ in this discharge, close to the Greenwald density of the ITER 9 MA steady-state scenario at 7.2 × 10^19^ m^−3^. The dedicated D_2_ gas puffing time trace is shown in vermillion in Fig. 2c. This approach ensures that there is a sufficient source of particles in the plasma, and it pushes the plasma density to a higher level, regardless of the change in the feedback gas (black line in Fig. 2c).

**Extended Data Fig. 2:**
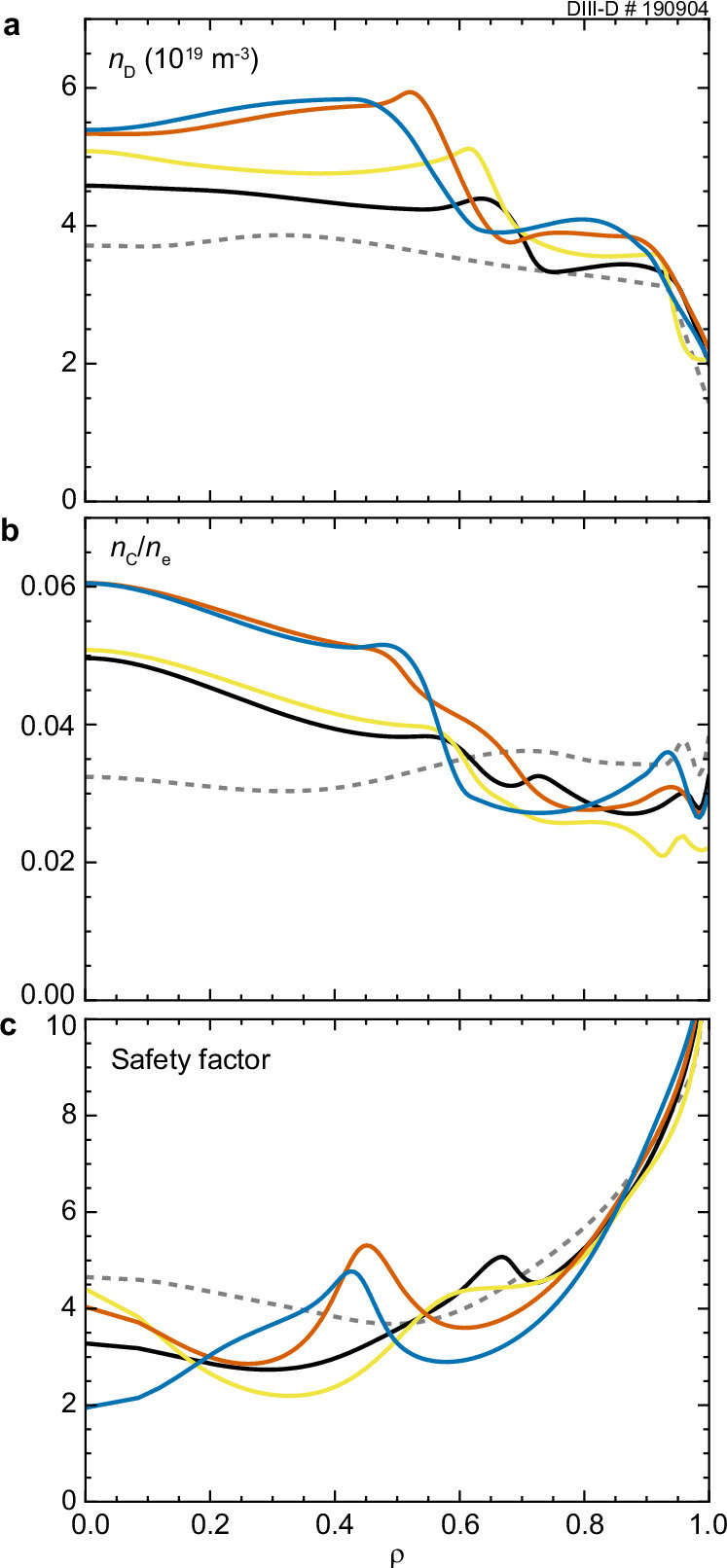
Additional profiles for DIII-D # 190904. Deuterium density profiles in (a), ratio between carbon density and electron density in (b) and safety factor profiles (q-profiles) in (c). Different color indicates the time slice shown in Fig. 2a. Additionally, profiles for a pre-ITB time slice (1.89 s) shown in gray dashed line are added.

Profiles of the temperature and density for electrons, deuterium (main ion) and carbon (main impurity) are shown in Fig. 2f–i and Extended Data Fig. 2a. The evolution of the on-axis densities for electrons, deuterium and carbon is displayed in Extended Data Fig. 1c. One can see that ITBs developed in all density channels. The increased deuterium density in this experiment suggests the promising application of this scenario in future FPPs, as it can attain a higher fuel density to give a higher fusion power. A related piece of experimental evidence is shown in Extended Data Fig. 1d. It is clear that with increased plasma density and energy confinement, the neutron rate, an indicator of fusion performance, increased substantially (67% higher, from 0.6 × 10^15^ to 1.0 × 10^15^ s^−1^) from 2 to 4.8 s, whereas the injected power (blue line in Extended Data Fig. 1e) was almost constant. Moreover, a very mild increase of the radiated power was observed in the very-high-density phase of the experiment (Extended Data Fig. 1e). The core radiated power as a fraction of the injected power increased from 10% to 20% as *f*_Gr_ increased from 0.7 to 1.1. The edge radiation remained about 25% of the injected power. Note that for either Bremsstrahlung radiation or impurity line emission, the radiated power was roughly proportional to the electron density squared. Therefore, some increase in the radiated power was expected even with the same impurity level, when the plasma density was increased significantly. Regarding the impurity behaviour, one can see a well-developed ITB at large radius in the carbon density profiles (Fig. 2i). Despite the ITB at large radius, the carbon density inside the ITB did not have a significant central peak, which would usually cause a large amount of core radiation and a reduction of core performance. The ratio between carbon density and electron density stayed around 4–5% during the evolution (Extended Data Fig. 2b). This is consistent with the well-controlled radiated power in the phase with *f*_Gr_ > 1.0.

The evolution of the safety factor profile (*q*-profile) is shown in Extended Data Fig. 2c. The figure shows the self-organized *q*-profile evolution, which reflects the change of the local bootstrap current density associated with the development of a large-radius ITB. The local minimum *q* (*q*_min_) in the outer half of the plasma was at *ρ* ≈ 0.6 for around 2*τ*_R_. *q*_min_ in this discharge stayed above 2.

When a density ITB built up over time and was sustained, the total pedestal pressure at *ρ* = 0.88 did not change significantly (Fig. 2d). However, other pedestal parameters and the ELM behaviour changed. At *f*_Gr_ < 0.8, typical standard H-mode pressure profiles and typical large type-I ELMs were observed (Fig. 2j,m). At 0.8 ≤ *f*_Gr_ < 1.0, pressure profiles with an ITB and compound ELMs emerged (Fig. 2k,n). Finally, pressure profiles with a large ITB and small ELMs dominated the *f*_Gr_ ≥ 1.0 phase (Fig. 2l,o). During the evolution, a decreased peak pedestal electron density (*n*_e,ped_) gradient, increased separatrix electron density (*n*_e,sep_) and decreased ratio between *n*_e,ped_ and *n*_e,sep_ were observed, as shown in Fig. 2d,e. These parameter evolutions are consistent with the favourable conditions needed to access the small-ELM regime discussed in the literature^29^. A more detailed modelling analysis of the pedestal for different ELM behaviours will be discussed later in this paper.

**Extended Data Fig. 3:**
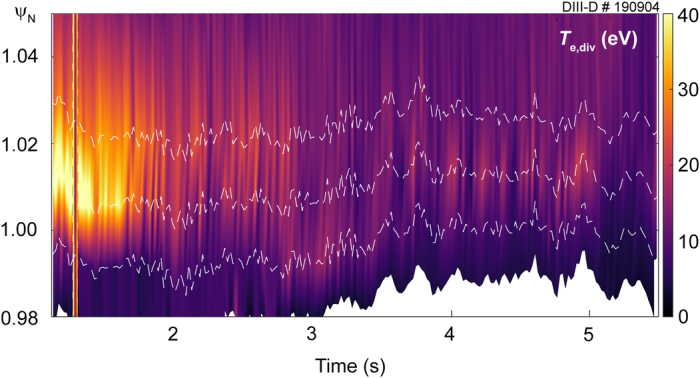
Spatial and temporal evolution of electron temperature at the divertor plates, measured by Langmuir probes. *ψ*_N_ is normalized poloidal flux. *ψ*_N_ < 1.0 locates within the private flux region. Color coding shows the measured *T*_e,div_. Dashed lines indicate the actual positions of Langmuir probes.

Although addressing the transient heat load is crucial, mitigating the stationary heat load is equally important for an FPP. Divertor detachment is widely considered to be a necessary solution for realizing an acceptable stationary heat load in the operation of future FPPs. Even without detachment-oriented impurity seeding, Extended Data Fig. 3 shows that the electron temperature at the divertor plates (*T*_e,div_) clearly reduced from over 35 eV (before 1.8 s) to 20–25 eV (1.8–2.8 s) and finally to 10–15 eV (after 2.8 s) in the *f*_Gr_ > 1.0 and *H*_98y2_ ≈ 1.5 phase, and there were small ELMs. The lowest *T*_e,div_ phase is consistent with the existence of an ITB at large radius. Although *T*_e,div_ ≤ 15 eV is not yet considered as divertor detachment (usually *T*_e,div_ < 10 eV), it already suggests that there would be mitigation of tungsten erosion under the experimental stationary heat load, if a tungsten wall had been used. Note that although the integration of full divertor detachment and high-confinement core has been achieved in previous DIII-D high-*β*_P_ experiments and reported^15,37^, the experimental approach and the operating parameter space were both different. The previous results used impurity seeding and *f*_Gr_ ≈ 0.9, which are not sufficient for FPP designs.

Therefore, the analysed typical DIII-D high-β_P_ discharge has demonstrated a sustained, accessible operating point in a present tokamak that integrates high normalized density and confinement quality, small ELMs and reduced divertor electron temperature, thus addressing the key requirements of FPP designs for simultaneous high-performance core and excellent core-edge integration.

**Fig. 3:**
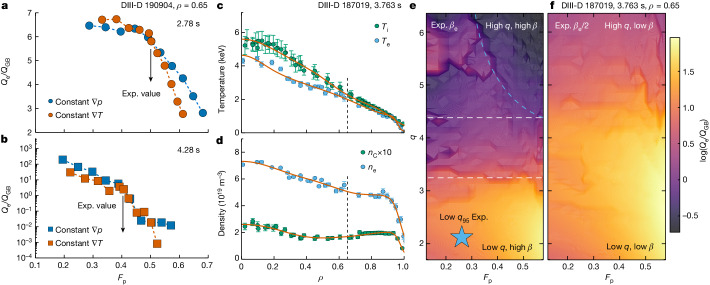
Transport modelling of the dependence of normalized electron turbulent heat flux on the normalized electron density gradient. **a**, Moderate *α*_MHD_ case from the high-*β*_P_ discharge in Fig. 2. *F*_p_ scan with the constant ∇*p* approach in blue and with the constant ∇*T* approach in vermillion. The experimental (Exp.) value of *F*_p_ is indicated by the black arrow. **b**, High *α*_MHD_ case from the high-*β*_P_ discharge in Fig. 2. Same colour coding as in **a**. **c**,**d**, Temperature (**c**) and density (**d**) profiles for the low-*q*_95_ H-mode case analysed in **e** and **f**. Dashed lines show the radial location for transport analysis. **e**,**f**, Two-dimensional scans of normalized electron turbulent heat flux on *F*_p_ and local *q* based on the low-*q*_95_ H-mode data shown in **c** and **d**. Full experimental *β*_e_ (**e**) and half experimental *β*_e_ (**f**). The experimental data point from the low-*q*_95_ discharge is indicated by a blue star in **e**.

To understand the physics that enables high confinement quality at high normalized density, we performed a gyro-fluid transport analysis using the TGLF code^38^ on the experimental data from the discharge shown in Fig. 2. Figure 3a,b shows the dependence of the normalized electron turbulent heat flux *Q*_e_/*Q*_GB_ (where *Q*_GB_ is the Gryo-Bohm heat flux) on the fractional contribution of the density gradient to the pressure gradient (*F*_p_ = *T*∇*n*/∇*p*) at mid-minor radius in the plasma. The gyro-fluid modelling indicates that when using either numerical approach to vary *F*_p_ (constant ∇*T* or constant ∇*p*), the decreasing trend of *Q*_e_ for increasing *F*_p_ is robust. A similar result was obtained for the ion energy transport. These results reveal an important feature in the high-*β*_P_ scenario, namely that anomalous turbulent transport, which leads to poor global confinement, can be reduced with a high density gradient, that is, a high density in the core with the pedestal density maintained below *n*_Gr_. This is consistent with the experimental observation of synergy between high confinement quality and high density. If *F*_p_ were increased by 30%, the normalized *Q*_e_ would decrease by a factor of 2 compared with the prediction at the experimental value, when the normalized pressure gradient *α*_MHD_ (approximately −*q*^2^/*B*_T,unit_^2^*R*d*p*/d*r*) was moderate (1.13), as shown in Fig. 3a. However, the reduction of the transport can be 2–3 orders of magnitude stronger when *α*_MHD_ is high (2.75) in the experimental equilibrium (Fig. 3b). Note that this finding is also consistent with the previous nonlinear gyro-kinetic theoretical modelling^39^, which found an extreme reduction in the transport coefficient when high *α*_MHD_ was combined with moderate density gradients. The underlying physics includes 1) the low drive of the ion-temperature-gradient turbulence at high density gradient (that is, there is a low ratio between the density gradient scale length and the ion temperature scale length (*η*_i_)), and 2) less effective coupling between trapped electrons and the trapped-electron-mode turbulence owing to the much narrower turbulence eigenfunction at high *α*_MHD_.

We also applied the same gyro-fluid transport analysis to a standard H-mode discharge to reveal the requirement for realizing the favourable conditions for low transport at high density. A low-*q*_95_ standard H-mode discharge (DIII-D 187019) with strong D_2_ gas puffing and high density was investigated. Compared with the high-*β*_P_ discharge discussed above, this discharge had the same heating power (9 MW), comparable line-averaged density (5.0–6.5 × 10^19^ m^−3^), slightly lower *β*_N_ (approximately 2.5), but much lower *q*_95_ (4 versus 8.5). This was because of a much higher *I*_p_ (1.3 versus 0.73 MA). Typical standard H-mode profiles are shown in Fig. 3c,d, which are different from the ITB profiles in Fig. 2. Figure 3e presents the transport analysis of a two-dimensional scan on *F*_p_ and local *q* at *ρ* = 0.65. The modelling uses the experimental *β*_e_ value. As illustrated by the horizontal dashed lines, the figure can be roughly divided into three regimes. At low local *q*, such as for the standard H-mode experimental data point, the modelling predicts high turbulent transport at high *F*_p_. This is consistent with the experimental observation of decreased *H*_98y2_ at high density in this discharge. At medium *q*, transport is predicted to be almost independent of *F*_p_. Finally, low transport at high *F*_p_ can be found in the high-*q* regime (top right corner of this figure highlighted by the blue dashed line). This example suggests that a minimum of the local *q* ≈ 4.4 is required to access this regime. Note that the analysed high-*β*_P_ case has local *q* ≈ 5.1. However, high local *q* alone is insufficient to access this regime. Figure 3f indicates the importance of sufficient *β*_e_, or the plasma pressure (*β*). Note that *β* changes accordingly in the modelling when scanning *β*_e_. The range of the two-dimensional scan is the same. However, this scan uses half of the experimental *β*_e_ in the modelling. As one can see, the results are significantly different. For most of the *q* values in the scan, high turbulent transport at high *F*_p_ is predicted. The favourable low transport at high *F*_p_ may still exist but probably requires very high local *q*, which is less realistic in present tokamak experiments or future machine designs.

In summary, the transport analysis suggests that the standard H-mode could access the favourable low-transport regime at high density, with the following necessary conditions: high local *q* and high plasma pressure *β*, which are two key components in the expression of *α*_MHD_. Thus, sufficient *α*_MHD_ is essential for realizing the favourable operating regime. As summarized in the literature^15,37,40^, α-stabilization is considered as the primary turbulence suppression physics in the high-*β*_P_ scenario, as it provides a reactor-relevant rotation-independent mechanism for high confinement^40^. On the other hand, given that *β*_P_ ∝ *β*_N_*q*_95_, high *q*_95_ and high *β*_N_ lead to high *β*_P_. Therefore, the high-*β*_P_ scenario is naturally an excellent candidate for pursing this goal.

**Fig. 4:**
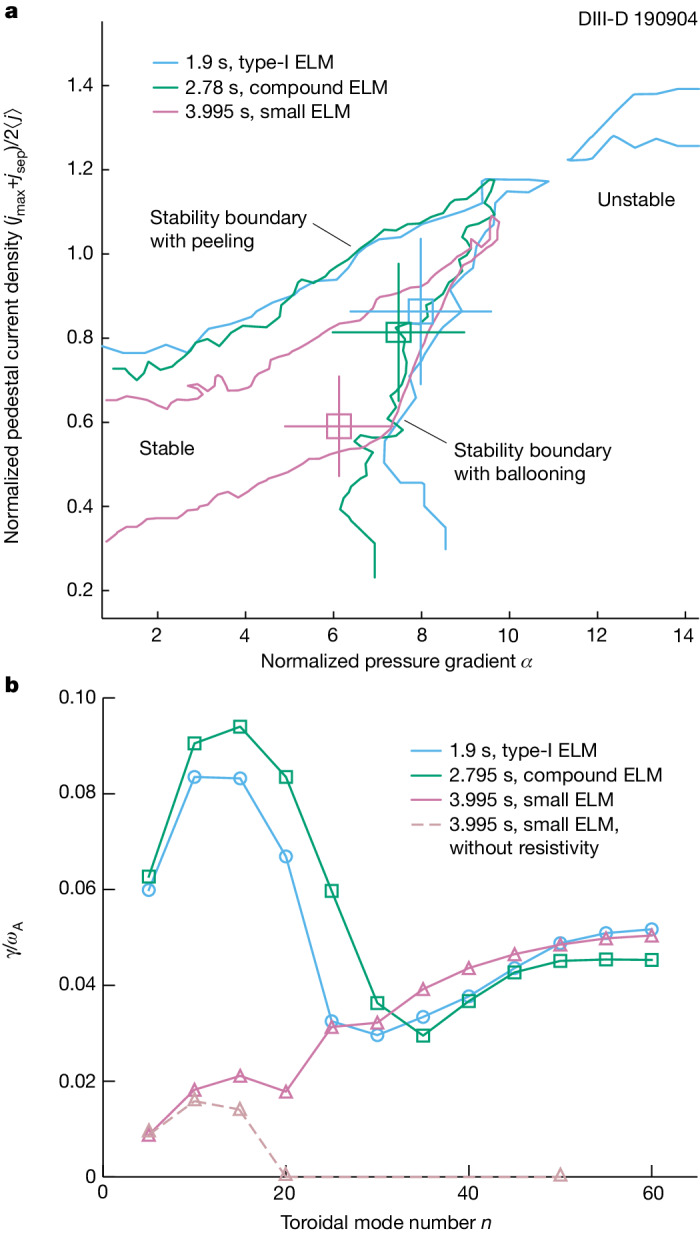
Pedestal modelling of the three types of ELM behaviours in DIII-D 190904. **a**,**b**, Results for the type-I ELM in blue, the compound ELM in green and the small ELM in violet. **a**, Pedestal stability versus normalized pedestal current density (*y* axis) and normalized pressure gradient at the pedestal peak gradient location (*x* axis). *j*_max_, *j*_sep_ and ⟨*j*⟩ are the maximum pedestal current density, the current density at the separatrix and the average current density in the pedestal region, respectively. Stability boundaries are shown as solid lines. Experimental points are indicated as open squares with error bars. **b**, Linear mode growth rate (normalized by Alfvén frequency, *ω*_A_) at different toroidal mode numbers.

We performed a pedestal analysis to evaluate the pedestal stability and understand the evolution of the ELM behaviour in the high-*β*_P_ discharge described in Fig. 2. The ELITE calculations^41^ shown in Fig. 4a predict the stability boundary for peeling–ballooning modes in the pedestal, for each of the three ELM states. In the type-I ELM state, the pedestal lies near the unstable ballooning region. Evolving to the small-ELM state, the experimental point moves along the ballooning boundary towards a lower pedestal pressure gradient and lower pedestal current density. Moving further away from the peeling boundary is consistent with the observation of no giant ELMs in the later phase. Modelling with BOUT++ (refs. ^42–44^) provides details on the instability growth rate in Fig. 4b. The dominant low-*n* peeling–ballooning mode was identified at *n* ≈ 10, which agrees with the ELITE result. The predicted low-*n* growth rate is smallest for small ELMs. BOUT++ modelling also resolves high-*n* resistive ballooning modes near the separatrix, when considering the plasma resistivity. It is clear that the high-*n* separatrix modes are dominant in the small-ELM case in contrast to other results in Fig. 4b. The modelling suggests that the high-*n* separatrix modes played an essential role in the observation of small ELMs in high-*β*_P_ plasmas.

In this paper, we have extended the operating space of a tokamak plasma towards a regime with simultaneous *f*_Gr_ up to 1.25 and *H*_98y2_ ≈ 1.3–1.8, using the high-*β*_P_ scenario in DIII-D. The achievement of entering this previously uncharted regime provides essential support to many attractive FPP designs all over the world. The increased deuterium density and neutron rate in the experiment confirm the promising application of this scenario for higher fusion performance in future FPPs. Unlike many previous high-density H-mode experiments, the high-*β*_P_ scenario uniquely features a synergy between high confinement quality and high density, especially around the Greenwald value. We have also elucidated the important role of α-stabilization in this achievement, showing that the favourable regime of low turbulent transport at high density is predicted and achieved only at relatively high local *q* and high *β*, namely for sufficient *α*_MHD_ at high *β*_P_. This successful experiment not only addresses a few of the key requirements on FPP core plasma parameters but also suggests a potential solution for core-edge integration by demonstrating sustained small ELMs together with *f*_Gr_ > 1.0 and *H*_98y2_ > 1.0. Realizing the small-ELM regime is understood as a combination of the reduced growth rate of low-*n* modes and the predominance of the high-*n* resistive ballooning mode near the separatrix because of the decreased peak density gradient in the pedestal, increased separatrix density and high *β*_P_. During the natural small-ELM phase with a high normalized density and confinement, the plasma is close to divertor detachment, which is believed to be the most promising solution for achieving steady-state plasma–wall-interactions in FPPs^37,45^. The natural proximity of detachment conditions shows the potential of a fully integrated scenario with high-performance core and cool edge. As the divertor detachment was not optimized in the discussed experiment, doing so will be important work for future experiments. Note that the compatibility of the high-*β*_P_ scenario with full divertor detachment has been demonstrated^37^. So far, neither a significant central peak in the density profile of the impurity (carbon) nor a significant increase in the core radiated power has been observed when the density is above the Greenwald value. Dedicated impurity transport experiments and modelling work are also under consideration for this operating regime. Fast-particle confinement is important for future FPPs. Experiments on the high-*β*_P_ scenario in DIII-D usually exhibit classical fast-ion transport. More discussion of previous results is presented in ‘DIII-D high-*β*_P_ experiments’ (Methods).

We fully appreciate that further work is needed to address other critical issues related to FPP compatibility, for example, operating with a metal wall and helium exhaust. On DIII-D, limited experiments with high-*β*_P_ plasmas operating with a divertor strike point on a (temporary) ring of tungsten tiles have shown promising results, with no significant degradation of core performance. However, to fully address the compatibility with a metal wall, we are collaborating closely with the Experimental Advanced Superconducting Tokamak (EAST) and Korea Superconducting Tokamak Advanced Research programme in the development of high-*β*_P_ scenarios so that we can exploit their metal wall and long-pulse operation capabilities. Long-pulse operation (over 10 s) will further address the alignment for steady-state *q*-profiles and pressure profiles with ITB in the high-*β*_P_ scenario. With regard to the helium exhaust, several review papers give favourable conclusions for high-*β*_P_ plasmas with ITBs in JT-60U^46,47^. The results for JT-60U high-*β*_P_ ITB plasmas show that the helium density in the core was controlled well and that no helium accumulation was observed, even with helium NBI for the core helium source. Moreover, the results also emphasize the importance of helium exhaust techniques, such as pumping, for controlling the helium content in the core.

Furthermore, there has been recent activity on extending the high-*β*_P_ scenario towards true long-pulse operation, including modelling work for EAST^48^, ITER^49,50^ and FPPs under design^10^. Depending on the design philosophy of each group, the high-*β*_P_ scenario can be applied in a wide range of FPP designs, from large conventional tokamaks^14^ to relatively small and compact devices^9,10^. One example from CAT-DEMO (Case D)^9^ shows a possible design point of an FPP using the high-*β*_P_ scenario: *R* = 4 m, *R*/*a* = 3.1, *B*_T_ = 7 T, *I*_p_ = 8.1 MA, *q*_95_ = 6.5, *f*_Gr_ = 1.3, *f*_Gr,ped_ = 1.0, *β*_N_ = 3.6, *H*_98y2_ = 1.51, fusion gain *Q* = 17.3 and output electric power 200 MWe. The experimental achievement and the increased understanding reported in this paper may open a potential avenue to an operating point for producing economically attractive fusion energy.

## Methods

### DIII-D tokamak

The DIII-D National Fusion Facility^51^ is a tokamak research device operated by General Atomic in San Diego, California, for the US Department of Energy. DIII-D is the largest magnetic fusion research facility in the USA. The DIII-D programme is focused on establishing the scientific basis for the optimization of the tokamak approach to fusion energy production, in part through the development of advanced steady-state operating scenarios. The major and minor radii of DIII-D are 1.66 and 0.67 m, respectively. The toroidal field is up to 2.2 T at the magnetic axis, and the plasma current is up to 2 MA. DIII-D has four high-power NBI systems with a total power of up to 20 MW. Unique features of the DIII-D NBI systems include: (1) two horizontally rotatable beamlines, providing a capability for switching the off-magnetic axis neutral beam current drive from the co plasma-current direction to the counter plasma-current direction and (2) two vertically movable beamlines that enable the selection of on- or off-magnetic axis plasma heating and current drive in the experiment. DIII-D has six operational gyrotrons for heating the electron cyclotron and driving the current in the plasma. Each gyrotron is designed for 1 MW continuous-wave operation for several seconds at a central frequency of 110 GHz. Steerable antennas offer flexible heating and current drive methods in the plasma, including mid-plane launchers and top launchers. DIII-D is also developing a helicon system and a lower-hybrid wave system for additional radio-frequency heating and current drive capabilities. The extensive diagnostic set and the sophisticated plasma control system support various plasma experiments on DIII-D.

### DIII-D high-*β*_P_ experiments

Many international tokamaks have investigated high-*β*_P_ scenarios^34,35,52–57^ ever since these scenarios were first proposed^14^ in 1990. High-*β*_P_ experiments were explored by DIII-D^58^ in the 2000s and have been extensively investigated since 2013 by the joint DIII-D/EAST research team^15^. An ITB at large minor radius is the signature of the high-*β*_P_ scenario in DIII-D. The latest physical understanding for developing such an ITB is through the reduced magnetic shear and sufficient α-stabilization effect^40,59^ (magnitude of *α*_MHD_). As *α*_MHD_ ∝ *q*^2^ ∝ *I*_p_^−2^, a relatively low plasma current and a broader current-density profile (having a higher *q* value and reduced magnetic shear at large radius) are beneficial for realizing ITB. DIII-D high-*β*_P_ experiments usually begin with a lower *I*_p_ flat top, aiming at *q*_95_ ≈ 10 in the first phase of the discharge. An *I*_p_ ramp-up or *B*_T_ ramp-down can be used in the later phase of the discharge to reduce the experimental *q*_95_ to a desired level, for example, 6–8. Edge perturbations, such as ELMs, active gas puffing and impurity injection have experimentally been found to trigger the formation of an ITB by creating a ‘low-(magnetic) shear detour’ at large radius, which gives access to the second stability regime. Experimentally, it has been found that an empirical *β*_P_ threshold of between 1.7 and 1.9 in DIII-D can sustain a strong ITB at large radius^15^. Regarding the plasma shape and configuration, a double-null configuration and an inverted ITER-similar true single-null shape have been successfully tested in experiments. Despite the high *q*_min_ (over 2) in experiments, the high-*β*_P_ scenario exhibits good fast-ion confinement, which is usually found to be classical. The present understanding^60,61^ indicates that: (1) The high-*β*_P_ plasmas have a shorter slowing-down time (because of the high density) and lower ∇*β*_fast_, where *β*_fast_ is the ratio of volume-averaged fast-ion pressure to the pressure of the toroidal magnetic field, which reduces the drive for Alfvénic modes. (2) The reverse-shear Alfvén eigenmodes are weaker or stable because the negative magnetic shear region is at higher radius, away from the peak of the fast-ion profile. Additionally, independent modelling of a high *q*_min_ (over 2) ITER 8 MA steady-state scenario (not the high-*β*_P_ scenario) found that there was negligible fast-ion loss because of a mismatch between the loss boundaries and the locations of the Alfvén eigenmodes^62^. A more detailed description of experimental waveform designs and a comprehensive review of the high-*β*_P_ scenario development on DIII-D in the last decade can be found in a review paper^15^.

### Diagnostics for profiles

A multi-pulsed high-resolution Thomson scattering system^63,64^ was used to measure the core electron density and temperature in the DIII-D experiments. The ion temperature and the carbon density profiles were measured by a charge-exchange recombination spectroscopy system for C^6+^ particles^65^. The measurements were arranged radially on the low-field side.

### Statistical analysis

Our statistical analysis used experimental data from DIII-D discharges during 2019–2022. We recorded data pairs of (*f*_Gr_, *H*_98y2_) from two time slices for each discharge: (1) the highest *H*_98y2_ and the corresponding *f*_Gr_ at the same time and (2) the highest *f*_Gr_ and the corresponding *H*_98y2_ at the same time, unless the two time slices were the same. Several filters were applied: *I*_p_ ≥ 0.55 MA, d*I*_p_/d*t* < 0.5 MA s^−1^, *P*_tot_ ≥ 5 MW, *W* ≥ 500 kJ and (d*W*/d*t*)/*P*_tot_ ≤ 0.1. Here, *W* is the total energy stored by the plasma and *P*_tot_ is the total heating power. A smoothing window of 40 ms was applied. The minimum and maximum *H*_98y2_ values were truncated at 0.5 and 2.5, respectively. More than 3,600 discharges from 2019–2022 were used in the analysis.

### Reconstruction of the kinetic equilibria

A multi-step workflow was used to add constraints on the pressure and plasma-current density in the equilibrium reconstruction to improve the accuracy of the reconstructed equilibrium. The workflow (for one iteration) has three parts: fitting the profile based on the existing equilibrium, calculating the power balance for the total pressure and plasma-current components, and reconstructing the equilibrium with pressure and current-density constraints. Usually, two or three iterations would be sufficient to produce high-quality equilibria for the transport study. When performing power balance calculation, NUBEAM^66^ was used for the NBI-driven current and fast-ion pressure calculation, and the Sauter model^67^ was used for the bootstrap current calculation. ONETWO^68^ was used to create the total pressure by combining the thermal pressure and fast-ion pressure and to provide the total plasma-current density by considering the external driven current, the bootstrap current and the calculated Ohmic current and by solving the poloidal field diffusion. The equilibrium was reconstructed with the EFIT code^69^.

### Gyro-fluid transport modelling

The TGLF code^38^ was used to calculate the turbulent heat fluxes in the high-*β*_P_ case and the low-*q*_95_ H-mode case. This modelling used a more recent saturation rule, SAT2 (ref. ^70^). Electromagnetic effects were included. The modelling took an experimental kinetic equilibrium from one time slice and focused on one radial location, for example, *ρ* = 0.65. The turbulent heat fluxes were predicted based on the local parameters of the selected time and radial location by taking contributions from several turbulent modes (low *k* and high *k*) into account. Quasi-neutrality was maintained when scanning the local density gradient *F*_p_, meaning that the density gradients for all species changed accordingly. When scanning the local *q*, other quantities that are not independent of *q* were scanned accordingly. When scanning *β*_e_, the entire plasma *β* was changed accordingly, as *T*_i_/*T*_e_ and *n*_i_/*n*_e_ were fixed in the modelling.

### Pedestal modelling

The ELITE code^41^ was used to calculate the growth rate of the peeling–ballooning mode instability, which is believed to limit the achievable pedestal height by triggering ELMs when the plasma crosses the instability boundary. ELITE uses reconstructed equilibria with pressure and current constraints (kinetic equilibria) as input. On the basis of the input, a set of equilibria were generated by independently varying the edge pressure and current in a separate ELITE calculation to obtain the peeling–ballooning boundary.

The reduced three-field fluid module under the BOUT++ framework^42–44^ was used to simulate the edge modes. The simulation evolved several physics parameters: perturbed pressure, magnetic flux and vorticity. The three-field module included not only basic peeling–ballooning physics but also non-ideal effects, such as ion diamagnetic drift, **E** × **B** drift, resistivity and so on. The simulation domain in the normalized poloidal flux was 0.80 < *ψ*_N_ < 1.05 and the grid resolution was *n*_ψ_ = 512 and *n*_y_ = 64. The kinetic equilibria were used as input. The Spitzer–Härm resistivity *η*_Sp_ was used by considering realistic plasma kinetic profiles. The hyper-resistivity was taken as a constant value *η*_H_ = 10^−16^ in the generalized Ohm’s law for current diffusion. The radial electric field (*E*_r_) profile calculated from the ion momentum balance equation was used in the simulation.

## Online content

Any methods, additional references, Nature Portfolio reporting summaries, source data, extended data, supplementary information, acknowledgements, peer review information; details of author contributions and competing interests; and statements of data and code availability are available at 10.1038/s41586-024-07313-3.

## Figures and Tables

**Extended Data Table 1:**
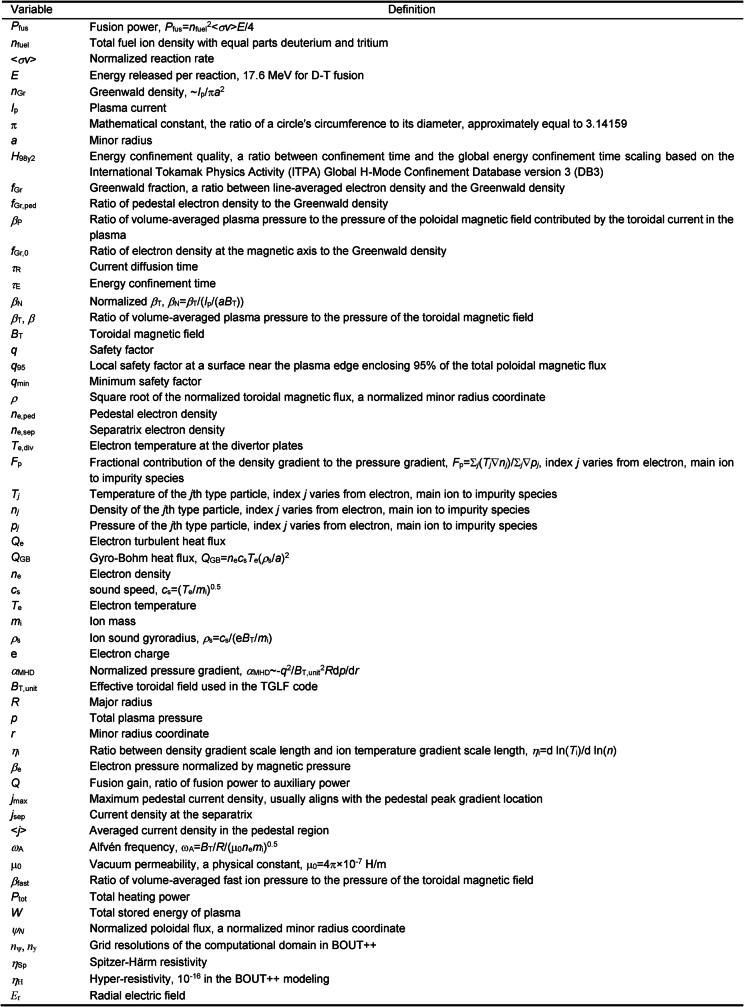
Terminology

## Data Availability

Raw data were generated by the DIII-D team. The data that support the findings of this study are available from the corresponding author upon request.

## Code availability

The computational codes used in the analysis of this paper are managed by General Atomics. They are available from the corresponding author upon reasonable request.
