# Author Correction: A high-density and high-confinement tokamak plasma regime for fusion energy

**DOI:** 10.1038/s41586-024-07545-3

**Published:** 2024-05-22

**Authors:** S. Ding, A. M. Garofalo, H. Q. Wang, D. B. Weisberg, Z. Y. Li, X. Jian, D. Eldon, B. S. Victor, A. Marinoni, Q. M. Hu, I. S. Carvalho, T. Odstrčil, L. Wang, A. W. Hyatt, T. H. Osborne, X. Z. Gong, J. P. Qian, J. Huang, J. McClenaghan, C. T. Holcomb, J. M. Hanson

**Affiliations:** 1 General Atomics, San Diego, CA USA; 2 Lawrence Livermore National Laboratory, Livermore, CA USA; 3 University of California San Diego, La Jolla, CA USA; 4 Plasma Science and Fusion Center, Massachusetts Institute of Technology, Cambridge, MA USA; 5 Princeton Plasma Physics Laboratory, Princeton University, Princeton, NJ USA; 6 Institute of Plasma Physics, Chinese Academy of Sciences, Hefei, China; 7 Department of Applied Mathematics and Applied Physics, Columbia University, New York, NY USA

**Keywords:** Magnetically confined plasmas, Nuclear fusion and fission

Correction to: *Nature* 10.1038/s41586-024-07313-3 Published online 24 April 2024

In the version of the article initially published, the first affiliation of A. Marinoni (University of California San Diego, La Jolla, CA, USA) was missing and has now been added to the HTML and PDF versions of the article.

